# Author Correction: Enhancing rice production sustainability and resilience via reactivating small water bodies for irrigation and drainage

**DOI:** 10.1038/s41467-023-40262-5

**Published:** 2023-07-27

**Authors:** Sisi Li, Yanhua Zhuang, Hongbin Liu, Zhen Wang, Fulin Zhang, Mingquan Lv, Limei Zhai, Xianpeng Fan, Shiwei Niu, Jingrui Chen, Changxu Xu, Na Wang, Shuhe Ruan, Wangzheng Shen, Menghan Mi, Shengjun Wu, Yun Du, Liang Zhang

**Affiliations:** 1 Hubei Provincial Engineering Research Center of Non-Point Source Pollution Control, Innovation Academy for Precision Measurement Science and Technology, Chinese Academy of Sciences, Wuhan, 430077 PR China; 2 Key Laboratory for Environment and Disaster Monitoring and Evaluation of Hubei, Wuhan, 430077 PR China; 3 University of Chinese Academy of Sciences, Beijing, 100049 PR China; 4 Institute of Agricultural Resources and Regional Planning, Chinese Academy of Agricultural Sciences, Beijing, 100081 PR China; 5 State Environmental Protection Key Laboratory of Soil Health and Green Remediation, Huazhong Agricultural University, Wuhan, 430070 PR China; 6 Interdisciplinary Research Center for Territorial Spatial Governance and Green Development, Huazhong Agricultural University, Wuhan, 430070 PR China; 7 Institute of Plant Protection, Soil and Fertilizer Sciences, Hubei Academy of Agricultural Sciences, Wuhan, 430064 PR China; 8 Chongqing Institute of Green and Intelligent Technology, Chinese Academy of Sciences, Chongqing, 400714 PR China; 9 Liaoning Academy of Agricultural Sciences, Shenyang, 110161 PR China; 10 Institute of Soil & Fertilizer and Resources & Environment, Jiangxi Academy of Agricultural Sciences, Nanchang, 330200 PR China

**Keywords:** Hydrology, Wetlands ecology, Environmental health

Correction to: *Nature Communications* 10.1038/s41467-023-39454-w, published online 26 June 2023

The original version of this Article included an incorrect Source Data file, in which the tab for Figure 2b had an error in row 13 column C where “0.0” appeared instead of “1.45”. The HTML has been updated to include a corrected version of the Source Data; the original incorrect version of the Source Data can be found as Supplementary Information associated with this Correction.

## Supplementary information

Incorrect Source Data

Correct Source Data

