# Supplementary material for: Enhancing rice production sustainability and resilience via reactivating small water bodies for irrigation and drainage
Source: Nat Commun. 2023 Jun 26;14:3794. doi: 10.1038/s41467-023-39454-w (PMC10293188; doi:10.1038/s41467-023-39454-w)
Supplement: Supplementary file 1 — Supplementary Information [file 41467_2023_39454_MOESM1_ESM.pdf]

Supplementary Information for

Enhancing rice production sustainability and resilience via reactivating small water bodies for irrigation and drainage

Sisi Li[1,2,3], Yanhua Zhuang[1,2,3], Hongbin Liu[4], Zhen Wang[5,6], Fulin Zhang[7], Mingquan Lv[8], Limei Zhai[4], Xianpeng Fan[7], Shiwei Niu[8], Jingrui Chen[9], Changxu Xu[9], Na Wang[8], Shuhe Ruan[1,2,3], Wangzheng Shen[1,2,3], Menghan Mi[1,2,3], Shengjun Wu[8], Yun Du[1,2,3], Liang Zhang[1,2,3] *

[1] Hubei Provincial Engineering Research Center of Non-Point Source Pollution Control, Innovation Academy for Precision Measurement Science and Technology, Chinese Academy of Sciences, Wuhan 430077, PR China

[2] Key Laboratory for Environment and Disaster Monitoring and Evaluation of Hubei, Wuhan 430077, PR China

[3] University of Chinese Academy of Sciences, Beijing 100049, PR China

[4] Institute of Agricultural Resources and Regional Planning, Chinese Academy of Agricultural Sciences, Beijing 100081, PR China

[5] State Environmental Protection Key Laboratory of Soil Health and Green Remediation, Huazhong Agricultural University, Wuhan 430070, PR China

[6] Interdisciplinary Research Center for Territorial Spatial Governance and Green Development, Huazhong Agricultural University, Wuhan 430070, PR China.

[7] Institute of Plant Protection, Soil and Fertilizer Sciences, Hubei Academy of Agricultural Sciences, Wuhan 430064, PR China

[8] Chongqing Institute of Green and Intelligent Technology, Chinese Academy of Sciences, Chongqing 400714, PR China

[9] Liaoning Academy of Agricultural Sciences, Shenyang 110161, PR China

[10] Institute of Soil & Fertilizer and Resources & Environment, Jiangxi Academy of Agricultural Sciences, Nanchang 330200, PR China

* **Corresponding Author at** Hubei Provincial Engineering Research Center of Non-Point Source Pollution Control, Innovation Academy for Precision Measurement Science and Technology, Chinese Academy of Sciences, Wuhan 430077, PR China.

Email address: lzhang@apm.ac.cn (L. Zhang)

Supplementary Figure/Table.

Supplementary Fig. 1 Province names and the locations of weather stations and typical surveyed irrigation drainage units in main rice production regions of China

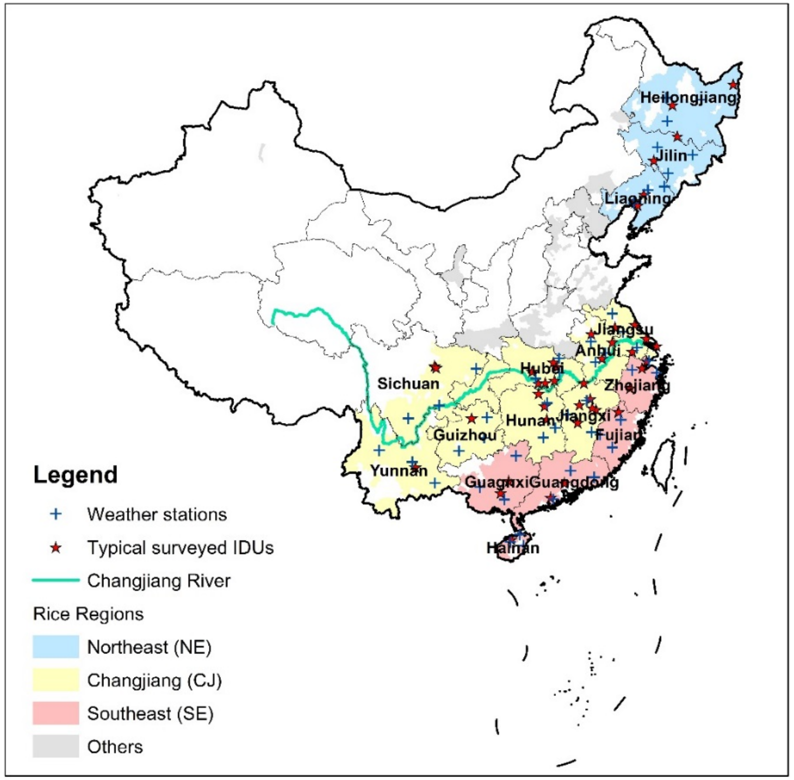

Supplementary Fig. 2 Workflow of data processing and model simulation. The spatial scales of the data are written in brackets.

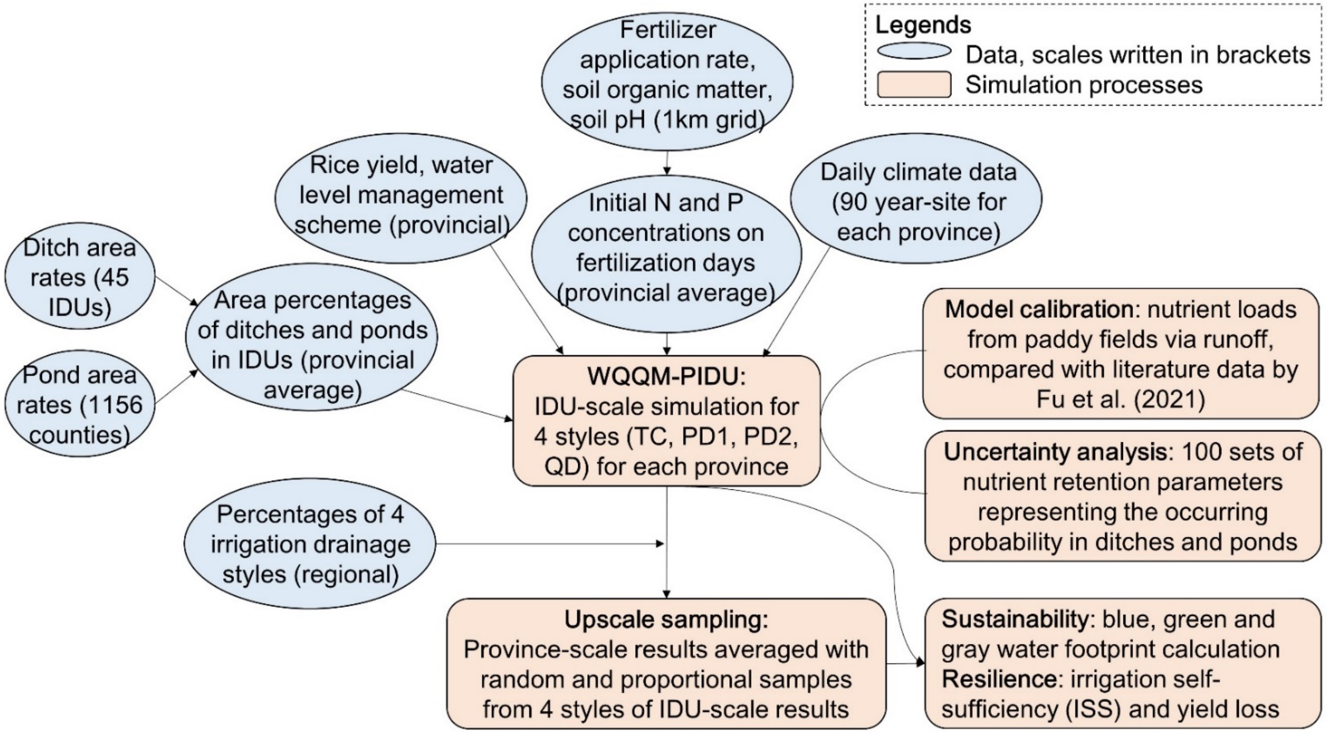

Supplementary Fig. 3 Comparison of simulated nutrient loads from rice fields via runoff in this study and by a published literature on the provincial scale. a) Comparison of TN loads, b) comparison of TP loads. For each province, 9000 simulation results representing the variability of 90 year-site realistic climate conditions and 100 sets of nutrient retention parameters were summarized. Provincial median values were used for comparison, and the gray error bars represent the interquartile ranges. P_{bias} is the percent bias, R^2 is the coefficient of determination for the provincial variations, and E_{NS} is the Nash-Sutcliffe coefficient for provincial variations, n is the number of provinces. Source data are provided as a Source Data file.

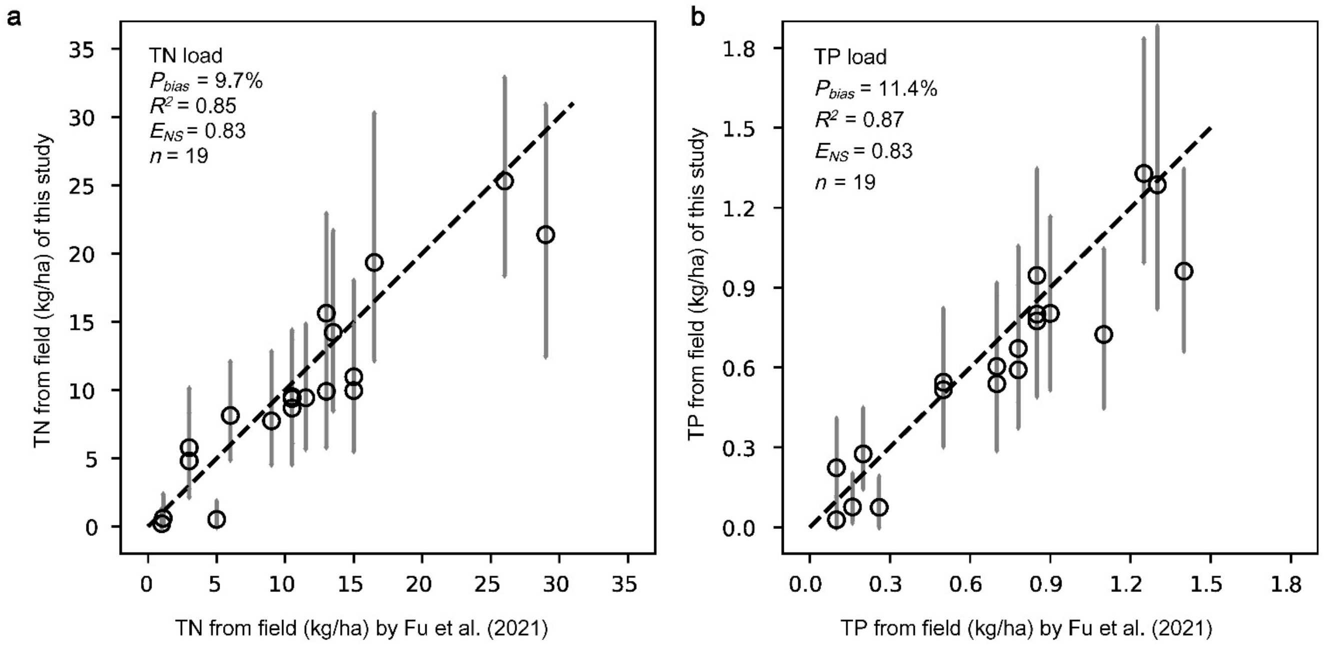

Supplementary Table 1. Policies affecting irrigation drainage system development in China

Start Year	Policy	Impact
1953	Agricultural water conservancy projects called by Ministry of Water Resources[1]	Rapid construction of small to medium size embankments and reservoirs, resulting in fast expansion of irrigated land during 1950s and 1970s.
1978	Land tenue reform to household; fiscal and taxation reform[2]	Pause of irrigation and drainage system construction due to unclear responsibility, lack of labor and financial investment during 1978-1987.
1988	Farmers' voluntary participation policy for agricultural water conservancy projects (Stop in 2004)[2]	Focus on the construction of medium to large size centralized irrigation districts, resulting in continuous increase of irrigated and drainage areas since 1988[1].
2002	Revision of the Water Law, with requirement of building a water-saving society[1]	Water-saving technology is encouraged in the construction of large irrigation district.
2013	National well-facilitated farmland construction overall plan by Ministry of agriculture and rural affairs[3]	Requires the construction of on-farm channels, ditches and small ponds to guarantee irrigation and drainage.
2019	Hydrologic connection and the construction of water beauty rural areas project proposed by Ministry of Water Resources and Ministry of Finance	National investment on hydrologic connection and the construction of water beauty rural areas in 167 counties of China since 2020, focusing on enhancing the flood regulation and environmental functions of small water bodies such as streams, small lakes and ponds.
2023	Agricultural non-point source pollution mitigation in national agricultural green development pilot counties proposed by Ministry of Agriculture and Rural Affairs	Encourage to use ditches and previous abandoned ponds to retain agriculture drainage water, in 128 national agricultural green development pilot counties.

Supplementary Table 2. Data description and the usage in this study

Data name	Data description	Scale	Usage
Statistic data on rice irrigation drainage system development in China	Number of Beitang, number of reservoirs, area of irrigated field and drainage field, population, rice yield, aquaculture pond from 1950 to 2020	Annual data in national and provincial scales	1. Analyze the evolution of rice irrigation drainage system in China, and its driving forces.<br>2. Rice yield data of each province is used in water footprint calculation along with WQQM-PIDU simulated nutrient loads.
Area percentages of ditches and ponds in IDUs	Area percentages of ditches were interpreted via remote sensing (Google Earth) images in 45 typical IDUs across 16 rice production provinces in China; Area percentages of ponds were extracted using a dataset of small water bodies in China by Lv et al. (2022)[4], ponds with a surface area less than 0.33 ha within 1156 rice production counties is used for calculation considering the availability of ponds for rice irrigation and drainage. An average area percentage is calculated for each province.	Current status in provincial scale	Input data of WQQM-PIDU model for the simulation of irrigation, drainage and nutrient loads.
Percentages of four irrigation and drainage management styles (as shown in Fig.1c) in current status	Questionnaire-based survey with experts from provincial institutes of agricultural sciences who have long-term field experience	Current status in three rice regions	Generate province and regional results from IDU scale results by randomly and proportionally sampling the IDU results of the four irrigation drainage styles.
Climate data	Daily rainfall, temperature, relative humidity, wind speed,	90 year-site of	1. Input data of WQQM-PIDU model for the

	sunshine hour for 30 years (1988-2017) from 3 climate stations in each of the 16 rice production provinces, collected from the China meteorological data service center.	daily data on provincial scale	simulation of irrigation, drainage and nutrient loads.<br>2. Calculate the aridity index (the rate of potential evapotranspiration to precipitation) on the rice growing season scale and the no-rain days before irrigation on the irrigation event scale.
Initial nitrogen and phosphorus concentrations on the fertilization days	Calculated with fertilizer application rate, soil pH, and soil organic matter data on 1km grid scales based on the relationship extracted from 76 literatures with 3486 data by Ruan et al (2022)[5] and averaged for each rice production province.	Averaged on provincial scale	Input data of WQQM-PIDU model for the simulation of nutrient concentration variation and nutrient loads.
Water level management scheme on rice field	Minimum suitable water level for rice, maximum suitable water level for rice and maximum tolerable water level for rice for rice growing periods for rice production provinces, collected by experts from provincial institutes of agricultural sciences.	Provincial scale	Input data of WQQM-PIDU model for the simulation of irrigation and drainage as well as nutrient loads.
Nutrient retention related parameters of ditches and ponds	100 sets of nutrient retention velocity (*vf_N and vf_P*, in cm/d) in nutrient spiraling theory and the equilibrium concentrations (*ENC0* and *EPC0*, in mg/L), generated by randomly sampling from a lognormal distribution representing the parameter occurring probability fitted by literature data by Shen et al. (2021)[6]		Input data of WQQM-PIDU model for uncertainty analysis considering the nutrient retention variability in ditches and ponds.

Supplementary Table 3. The occurring rate of differently managed irrigation and drainage units in current systems and for system redesign scenarios incorporating more decentralized management utilizing small water bodies. TC refers to totally centralized, PD1 refers to partly decentralized 1, PD2 refers to partly decentralized 2, QD refers to quasi-decentralized. The four management styles are depicted in Fig. 1c.

**Rice region**	**Northeast rice region**				**Changjiang River basin rice region**				**Southeastern coastal rice region**			
**Management styles**	**TC**	**PD1**	**PD2**	**QD**	**TC**	**PD1**	**PD2**	**QD**	**TC**	**PD1**	**PD2**	**QD**
**Current status**	0.75	0.25	0.00	0.00	0.76	0.04	0.16	0.04	0.60	0.26	0.13	0.02
**Recycling irrigation 1**	0.66	0.34	0.00	0.00	0.67	0.14	0.14	0.06	0.52	0.33	0.11	0.04
**Recycling irrigation 2**	0.56	0.44	0.00	0.00	0.57	0.23	0.12	0.08	0.45	0.40	0.10	0.05
**Recycling irrigation 3**	0.47	0.53	0.00	0.00	0.48	0.33	0.10	0.10	0.37	0.48	0.08	0.07
**Recycling irrigation 4**	0.38	0.63	0.00	0.00	0.38	0.42	0.08	0.12	0.30	0.55	0.06	0.09
**Recycling irrigation 5**	0.28	0.72	0.00	0.00	0.29	0.52	0.06	0.14	0.22	0.63	0.05	0.10
**Recycling irrigation 6**	0.19	0.81	0.00	0.00	0.19	0.61	0.04	0.16	0.15	0.70	0.03	0.12
**Recycling irrigation 7**	0.09	0.91	0.00	0.00	0.10	0.71	0.02	0.18	0.07	0.78	0.02	0.13
**Recycling irrigation 8**	0.00	1.00	0.00	0.00	0.00	0.80	0.00	0.20	0.00	0.85	0.00	0.15
**Pond reconnection 1**	0.00	0.88	0.00	0.13	0.00	0.70	0.00	0.30	0.00	0.74	0.00	0.26
**Pond reconnection 2**	0.00	0.75	0.00	0.25	0.00	0.60	0.00	0.40	0.00	0.64	0.00	0.36
**Pond reconnection 3**	0.00	0.63	0.00	0.38	0.00	0.50	0.00	0.50	0.00	0.53	0.00	0.47
**Pond reconnection 4**	0.00	0.50	0.00	0.50	0.00	0.40	0.00	0.60	0.00	0.43	0.00	0.58
**Pond reconnection 5**	0.00	0.38	0.00	0.63	0.00	0.30	0.00	0.70	0.00	0.32	0.00	0.68
**Pond reconnection 6**	0.00	0.25	0.00	0.75	0.00	0.20	0.00	0.80	0.00	0.21	0.00	0.79
**Pond reconnection 7**	0.00	0.13	0.00	0.88	0.00	0.10	0.00	0.90	0.00	0.11	0.00	0.89
**Pond reconnection 8**	0.00	0.00	0.00	1.00	0.00	0.00	0.00	1.00	0.00	0.00	0.00	1.00
**Pond construction 1**	0.00	0.00	0.00	1.00	0.00	0.00	0.00	1.00	0.00	0.00	0.00	1.00
**Pond construction 2**	0.00	0.00	0.00	1.00	0.00	0.00	0.00	1.00	0.00	0.00	0.00	1.00
**Pond construction 3**	0.00	0.00	0.00	1.00	0.00	0.00	0.00	1.00	0.00	0.00	0.00	1.00
**Pond construction 4**	0.00	0.00	0.00	1.00	0.00	0.00	0.00	1.00	0.00	0.00	0.00	1.00
**Pond construction 5**	0.00	0.00	0.00	1.00	0.00	0.00	0.00	1.00	0.00	0.00	0.00	1.00
**Pond construction 6**	0.00	0.00	0.00	1.00	0.00	0.00	0.00	1.00	0.00	0.00	0.00	1.00
**Pond construction 7**	0.00	0.00	0.00	1.00	0.00	0.00	0.00	1.00	0.00	0.00	0.00	1.00
**Pond construction 8**	0.00	0.00	0.00	1.00	0.00	0.00	0.00	1.00	0.00	0.00	0.00	1.00

Supplementary Table 4. Ditch and pond parameters in current status, recycling irrigation and pond reconnection scenarios. HB: Hubei, AH: Anhui, GZ: Guizhou, JS: Jiangsu, SC: Sichuan, YN: Yunnan, HuN: Hunan, JX: Jiangxi, FJ: Fujian, GD: Guangdong, GX: Guangxi, HaN: Hainan, ZJ: Zhejiang, HLJ: Heilongjiang, JL: Jilin, LN: Liaoning

**Rice region**	**Changjiang River basin rice region**								**Southeastern coastal rice region**					**Northeast rice region**		
**Scenarios**	**Current status, Recycling irrigation, pond reconnection**															
**Provinces**	**HB**	**AH**	**GZ**	**JS**	**SC**	**YN**	**HuN**	**JX**	**FJ**	**GD**	**GX**	**HaN**	**ZJ**	**HLJ**	**JL**	**LN**
**Area rate of ditches and ponds (%)**	7.0	7.3	2.3	7.5	3.0	2.6	6.3	7.1	6.0	5.2	4.2	9.1	5.1	5.0	4.0	5.3
**Water storage volume (mm)**	56	61	16	66	32	20	60	58	42	46	36	82	42	32	27	32
**1st level ditch**																
depth (m)	0.8	0.8	0.8	0.8	0.8	0.8	0.8	0.8	0.8	0.8	0.8	0.8	0.8	0.5	0.5	0.5
surface wide (m)	1.5	1.5	1.5	1.5	1.5	1.5	1.5	1.5	1.5	1.5	1.5	1.8	1.5	1.5	1.5	1.5
bottom wide (m)	0.3	0.3	0.7	0.3	0.7	0.7	0.3	0.3	0.3	0.3	0.3	0.6	0.3	0.3	0.3	0.3
side slope	0.75	0.75	0.50	0.75	0.50	0.50	0.75	0.75	0.75	0.75	0.75	0.75	0.75	1.20	1.20	1.20
interval (m)	60	50	100	55	150	100	80	50	55	80	100	50	75	60	80	50
**2nd level ditch**																
depth (m)	1.0	1.0	1.0	1.0	1.0	1.0	1.0	1.0	1.0	1.0	1.0	1.0	1.0	1.2	1.2	1.2
surface wide (m)	2.8	3.0	2.0	3.5	2.0	2.0	2.8	3.5	3.0	2.8	2.5	3.5	2.8	6.8	6.8	6.8
bottom wide (m)	0.8	1.0	0.8	1.5	0.8	0.8	0.8	1.5	1.0	0.8	0.9	1.5	0.8	3.2	3.2	3.2
side slope	1.00	1.00	0.60	1.00	0.60	0.60	1.00	1.00	1.00	1.00	0.80	1.00	1.00	1.50	1.50	1.50
interval (m)	80	100	350	100	320	300	120	110	100	150	150	85	140	260	350	260
**Pond**																
depth (m)	1.5	1.5	1.5	1.5	1.5	1.5	1.5	1.5	1.5	1.5	1.5	1.5	1.5	1.5	1.5	1.5
side slope	1.25	1.25	1.25	1.25	1.25	1.25	1.25	1.25	1.25	1.25	1.25	1.25	1.25	1.25	1.25	1.25
area rate of pond (%)	1.4	1.7	0.2	1.8	1.5	0.5	2.3	1.3	0.6	1.7	1.1	2.1	1.3	0.1	0.3	0.1

Supplementary Table 5. Area percentage of ponds (%) under pond construction scenarios with other ditch and pond parameters unchanged. HB: Hubei, AH: Anhui, GZ: Guizhou, SC: Sichuan, YN: Yunnan, HuN: Hunan, JX: Jiangxi, FJ: Fujian, GD: Guangdong, GX: Guangxi, ZJ: Zhejiang, HLJ: Heilongjiang, JL: Jilin, LN: Liaoning

**Rice Region**	**Changjiang River basin rice region**							**Southeastern coastal rice region**				**Northeast rice region**		
**Provinces**	**HB**	**AH**	**GZ**	**SC**	**YN**	**HuN**	**JX**	**FJ**	**GD**	**GX**	**ZJ**	**HLJ**	**JL**	**LN**
**Pond construction 1**	1.45	1.81	1.01	2.12	1.21	2.57	1.38	0.84	2.03	1.66	1.67	0.34	0.83	0.26
**Pond construction 2**	1.50	1.91	1.77	2.77	1.93	2.80	1.42	1.12	2.40	2.16	2.06	0.59	1.37	0.43
**Pond construction 3**	1.55	2.00	2.52	3.41	2.63	3.02	1.45	1.39	2.76	2.66	2.44	0.83	1.90	0.60
**Pond construction 4**	1.61	2.10	3.26	4.05	3.33	3.25	1.49	1.66	3.13	3.16	2.83	1.07	2.43	0.76
**Pond construction 5**	1.66	2.20	3.98	4.67	4.01	3.47	1.52	1.93	3.49	3.65	3.21	1.31	2.95	0.93
**Pond construction 6**	1.71	2.29	4.69	5.29	4.69	3.69	1.56	2.20	3.85	4.13	3.58	1.55	3.47	1.09
**Pond construction 7**	1.76	2.39	5.40	5.90	5.35	3.91	1.60	2.47	4.20	4.61	3.95	1.79	3.98	1.26
**Pond construction 8**	1.82	2.48	6.09	6.50	6.01	4.13	1.63	2.73	4.55	5.09	4.32	2.03	4.48	1.42

Supplementary References:

1. Liu J.G., Zang C.F., Tian S.Y., Liu J.G., Yang H., Jia S.F., You L.Z., Liu B., Zhang M., Water conservancy projects in China: Achievements, challenges and way forward. *Glob. Environ. Change-Human Policy Dimens*. **23**(3), 633-643 (2013).
2. Zhang C., Li D., Thinking on the construction of agricultural water conservancy in the new period. *China rural Water Conserv. Hydropower* (07), 1-3 (2009). (in Chinese)
3. Minstry of Agriculture and Rural Affairs of the People's Republic of China, National well-facilitated farmland construction plan (2021-2030). Beijing, China (2021). (in Chinese)
4. Lv M., Wu S., Ma M., Huang P., Wen Z., Chen J. Small water bodies in China: Spatial distribution and influencing factors. *Sci. China Earth Sci.*, **65**(8): 1431–1448 (2022). (in Chinese)
5. Ruan S., Zhuang Y., Zhang L., Li S., Chen J., Wen W., Zhai L., Liu H., Du Y., Improved estimation of nitrogen dynamics in paddy surface water in China. *J. Environ. Manage*. **312**, 114932 (2022).
6. Shen W., Li S., Mi M., Zhuang Y., Zhang L., What makes ditches and ponds more efficient in nitrogen control? *Agric. Ecosyst. Environ*. **314**, 107409 (2021).
