# Supplementary material for: Enhancing rice production sustainability and resilience via reactivating small water bodies for irrigation and drainage
Source: Nat Commun. 2023 Jun 26;14:3794. doi: 10.1038/s41467-023-39454-w (PMC10293188; doi:10.1038/s41467-023-39454-w)
Supplement: Supplementary file 2 — Reporting Summary [file 41467_2023_39454_MOESM2_ESM.pdf]

nature portfolio

Corresponding author(s): Liang Zhang

Last updated by author(s): Jun 6, 2023

Reporting Summary

Nature Portfolio wishes to improve the reproducibility of the work that we publish. This form provides structure for consistency and transparency in reporting. For further information on Nature Portfolio policies, see our Editorial Policies and the Editorial Policy Checklist.

Statistics

For all statistical analyses, confirm that the following items are present in the figure legend, table legend, main text, or Methods section.

n/a	Confirmed	
☐	☒	The exact sample size (*n*) for each experimental group/condition, given as a discrete number and unit of measurement
☒	☐	A statement on whether measurements were taken from distinct samples or whether the same sample was measured repeatedly
☒	☐	The statistical test(s) used AND whether they are one- or two-sided<br>*Only common tests should be described solely by name; describe more complex techniques in the Methods section.*
☒	☐	A description of all covariates tested
☒	☐	A description of any assumptions or corrections, such as tests of normality and adjustment for multiple comparisons
☐	☒	A full description of the statistical parameters including central tendency (e.g. means) or other basic estimates (e.g. regression coefficient) AND variation (e.g. standard deviation) or associated estimates of uncertainty (e.g. confidence intervals)
☒	☐	For null hypothesis testing, the test statistic (e.g. *F*, *t*, *r*) with confidence intervals, effect sizes, degrees of freedom and *P* value noted<br>*Give P values as exact values whenever suitable.*
☒	☐	For Bayesian analysis, information on the choice of priors and Markov chain Monte Carlo settings
☒	☐	For hierarchical and complex designs, identification of the appropriate level for tests and full reporting of outcomes
☒	☐	Estimates of effect sizes (e.g. Cohen's *d*, Pearson's *r*), indicating how they were calculated

Our web collection on statistics for biologists contains articles on many of the points above.

Software and code

Policy information about availability of computer code

Data collection	The area percentages of ditches for surveyed irrigation drainage units are extracted by visual interpretation using Google Earth pro v7.3, other data collection did not use specific softwares.
Data analysis	The WQQM-PIDU model used in this study is version 2.0, this model code and the analysis scripts for water footprint and irrigation self-sufficiency calculation are available in GitHub: https://github.com/Li-Sisi2020/WQQM-PIDU with a DOI: https://zenodo.org/badge/latestdoi/538525282.

For manuscripts utilizing custom algorithms or software that are central to the research but not yet described in published literature, software must be made available to editors and reviewers. We strongly encourage code deposition in a community repository (e.g. GitHub). See the Nature Portfolio guidelines for submitting code & software for further information.

Data

Policy information about availability of data

All manuscripts must include a data availability statement. This statement should provide the following information, where applicable:

- Accession codes, unique identifiers, or web links for publicly available datasets
- A description of any restrictions on data availability
- For clinical datasets or third party data, please ensure that the statement adheres to our policy

Source data are provided with this paper. Statistical data on irrigation drainage system development in China are collected mainly from the national bureau of statistics (https://data.stats.gov.cn); Climate data are collected from the China meteorological data service center (https://data.cma.cn). Other data supporting the

main findings of this study and the important parameters used in the modeling analysis can be found in the Supplementary Information and the Supplementary Data files.

Human research participants

Policy information about studies involving human research participants and Sex and Gender in Research.

Reporting on sex and gender	*Use the terms sex (biological attribute) and gender (shaped by social and cultural circumstances) carefully in order to avoid confusing both terms. Indicate if findings apply to only one sex or gender; describe whether sex and gender were considered in study design whether sex and/or gender was determined based on self-reporting or assigned and methods used. Provide in the source data disaggregated sex and gender data where this information has been collected, and consent has been obtained for sharing of individual-level data; provide overall numbers in this Reporting Summary. Please state if this information has not been collected. Report sex- and gender-based analyses where performed, justify reasons for lack of sex- and gender-based analysis.*
Population characteristics	*Describe the covariate-relevant population characteristics of the human research participants (e.g. age, genotypic information, past and current diagnosis and treatment categories). If you filled out the behavioural & social sciences study design questions and have nothing to add here, write "See above."*
Recruitment	*Describe how participants were recruited. Outline any potential self-selection bias or other biases that may be present and how these are likely to impact results.*
Ethics oversight	*Identify the organization(s) that approved the study protocol.*

Note that full information on the approval of the study protocol must also be provided in the manuscript.

Field-specific reporting

Please select the one below that is the best fit for your research. If you are not sure, read the appropriate sections before making your selection.

☐ Life sciences ☐ Behavioural & social sciences ☒ Ecological, evolutionary & environmental sciences

For a reference copy of the document with all sections, see nature.com/documents/nr-reporting-summary-flat.pdf

Ecological, evolutionary & environmental sciences study design

All studies must disclose on these points even when the disclosure is negative.

Study description	First, a systematic survey was conducted to figure out the evolution and current status of irrigation drainage systems in rice regions of China. Then, a simulation analysis using WQQM-PIDU model was conducted to analyze the impacts of differently managed systems on system sustainability and resilience. Last, three system redesign approaches were proposed to reactivate small water bodies in rice irrigation and drainage. The potential benefits and trade-off of the redesigned system were quantified. Benefits were quantified as the reduced water footprints, increased irrigation self-sufficiency and reduced yield loss under dry climate. The trade-off was quantified as the percentages of land occupation. In addition, these benefits were tested in field studies by comparing the irrigation drainage units (IDUs) with these approaches and the control IDUs with current management. The robustness of the simulation results is guaranteed by both model calibration and uncertainty quantification. First, the model was calibrated by comparing the simulated nutrient export from paddy fields via runoff with published literature results, with indicators including the percent bias and the coefficient of determination. Second, nutrient retention related parameters of the WQQM-PIDU are the main uncertainty source so that 100 sets of these parameters generated from a log-normal probability distribution fitted by literature reported values were used to quantify the uncertainty in terms of gray water footprints.
Research sample	To generate an overall picture of the impacts of differently managed irrigation drainage systems representing various climate conditions and nutrient retention capability, WQQM-PDIU model was run for 9000 times for each of the four management styles in each rice-growing province as a function of 90 sets of climate data and 100 sets of nutrient retention parameters. The 90 sets of climate data represent three typical sites in a province and 30 years of climate data, which is enough to represent climate variability in rice regions of China; the 100 sets of nutrient retention parameters represent a wide range of the variability in nutrient retention capabilities of ditches and ponds, which are generated from a log-normal probability distribution fitted by literature reported values of these parameters. Then, the overall results for each province is calculated by the average value of the proportionally sampled results from the four management styles according to their occurrence percentages in current status and future scenarios; while the overall results for China is the rice yield weighted average value of all the main rice-growing provinces. This proportional sampling is rational because it represents the realistic percentages of differently-managed sub-systems existed in current China as we surveyed.
Sampling strategy	The sampling strategy is Monte Carlo sampling, which is random from a wide possible values. A total of 9000 samples were selected proportional to the percentages of the four irrigation drainage unit-scale management styles existed in current China and for proposed scenarios, which is enough to represent variability related to climate, nutrient retention capability, etc.
Data collection	The data of this study include: (1) the statistic data on irrigated fields, drainage fields and the number of tradtional decentralized irrigation drainage systems (Beitangs), which is collected by authors from national statistic database and yearbooks. (2) The structure and management styles of current irrigation drainage units (IDUs) by remote sensing survey and expert survey by authors. Specifically

	for the area percentages of ponds in IDUs, a recently published dataset of small water bodies in China is used to extract 2.75 million small ponds in rice growing regions and the area percentages are analyzed for 1156 rice-growing counties. (3) Climate data from the China meteorological data service center collected by authors; (4) rice field management practices and related parameters used in model analysis collected and determined by authors; (5) nutrient processing parameters used in the model analysis collected from literatures.
Timing and spatial scale	The timing of the climate data used for simulation analysis cover 30 years from 1988 to 2017, which make the results representative to various weather conditions, from dry to wet.<br>The spatial scale of this study cover all main rice regions in China, including three rice regions and 16 provinces. For each province, analysis is conducted to represent the typical irrigation drainage unit structure and management styles, the typical climate condition, and typical agricultural management practices.
Data exclusions	No data was excluded in this simulation analysis.
Reproducibility	Our study is an integrated study mainly based on model simulation with climate, nutrient processing and agricultural management data. Our results can be reproduced when following the described methods and data.
Randomization	This is not relevant to our study because our work is not an "experimental" study but an integrated data analysis. We collected enough data to represent various climate variation, nutrient processing variation and agricultural management practices.
Blinding	Blinding is not applicable to our study, because we collected enough data to represent various climate variation, nutrient processing variation and agricultural management practices.

Did the study involve field work? ☒ Yes ☐ No

Field work, collection and transport

Field conditions	The three field observation sites are located in three different rice-growing regions with various climate conditions and management practices: (1) Anhui, Hubei site is in the Changjiang one-season rice region, the annual precipitation is about 1100 mm, the average temperature is 15.9 °C; (2) Gaoan Jiangxi site is in the Changjiang two-season rice region, the average annual precipitation is about 1600 mm, the average temperature is 18.9 °C; (3) Panjin, Liaoning site is in the Northeast rice region, the average annual precipitation is about 640 mm, the average temperature is about 10.9 °C.
Location	The field observations are conducted in three typical rice-growing areas: Anlu, Hubei (113.6779E, 31.3364N, 46m), Gaoan, Jiangxi (115.1256E, 28.2555N, 37m) and Panjin, Liaoning (122.2504E, 41.1724N, 4m)
Access & import/export	The rice yield, water quantity and nutrient concentrations exported from the tested and control irrigation drainage units are observed and tested by third-party organizations which hold related certificates. Their sampling and measurement methods obey the requirements of national rules in China.
Disturbance	Field observations can reflect limited climate conditions. The observations were conducted in the whole rice season in 2020, a relatively wet year. We used simulation analysis to represent more climate conditions.

Reporting for specific materials, systems and methods

We require information from authors about some types of materials, experimental systems and methods used in many studies. Here, indicate whether each material, system or method listed is relevant to your study. If you are not sure if a list item applies to your research, read the appropriate section before selecting a response.

Materials & experimental systems

n/a	Involved in the study	
☒	☐	Antibodies
☒	☐	Eukaryotic cell lines
☒	☐	Palaeontology and archaeology
☒	☐	Animals and other organisms
☒	☐	Clinical data
☒	☐	Dual use research of concern

Methods

n/a	Involved in the study	
☒	☐	ChIP-seq
☒	☐	Flow cytometry
☒	☐	MRI-based neuroimaging
