# Supplementary material for: Enhancing rice production sustainability and resilience via reactivating small water bodies for irrigation and drainage
Source: Nat Commun. 2023 Jun 26;14:3794. doi: 10.1038/s41467-023-39454-w (PMC10293188; doi:10.1038/s41467-023-39454-w)
Supplement: Supplementary file 3 — Description of Additional Supplementary Files [file 41467_2023_39454_MOESM3_ESM.pdf]

File Name: Supplementary Data 1

Description: The 100 parameter sets for nutrient retention of ditches and ponds generated with Monte Carlo sampling. *vf_N* and *vf_P* refers to nitrogen and phosphorus retention velocity in nutrient spiraling theory, in cm/d; *ENC0* and *EPC0* refers to equilibrium nitrogen and phosphorus concentrations used in WQQM-PIDU model.
